# Identification of a Steroid Hormone-Associated Gene Signature Predicting the Prognosis of Prostate Cancer through an Integrative Bioinformatics Analysis

**DOI:** 10.3390/cancers14061565

**Published:** 2022-03-19

**Authors:** Yo-Liang Lai, Chia-Hsin Liu, Shu-Chi Wang, Shu-Pin Huang, Yi-Chun Cho, Bo-Ying Bao, Chia-Cheng Su, Hsin-Chih Yeh, Cheng-Hsueh Lee, Pai-Chi Teng, Chih-Pin Chuu, Deng-Neng Chen, Chia-Yang Li, Wei-Chung Cheng

**Affiliations:** 1 Graduate Institute of Biomedical Science, China Medical University, Taichung 40403, Taiwan; yolianglai@gmail.com; 2 Department of Radiation Oncology, China Medical University Hospital, Taichung 40403, Taiwan; 3 Research Center for Cancer Biology, China Medical University, Taichung 40403, Taiwan; b881642@gmail.com (C.-H.L.); Demicho.0111@gmail.com (Y.-C.C.); 4 Department of Medical Laboratory Science and Biotechnology, Kaohsiung Medical University, Kaohsiung 80708, Taiwan; shuchiwang@kmu.edu.tw; 5 Department of Urology, School of Medicine, College of Medicine, Kaohsiung Medical University, Kaohsiung 80708, Taiwan; shpihu73@gmail.com (S.-P.H.); patrick1201.tw@yahoo.com.tw (H.-C.Y.); 6 Department of Urology, Kaohsiung Medical University Hospital, Kaohsiung Medical University, Kaohsiung 80708, Taiwan; hsueh612@hotmail.com; 7 Graduate Institute of Clinical Medicine, College of Medicine, Kaohsiung Medical University, Kaohsiung 80708, Taiwan; 8 Ph.D. Program in Environmental and Occupational Medicine, College of Medicine, Kaohsiung Medical University, Kaohsiung 80708, Taiwan; 9 Department of Pharmacy, China Medical University, Taichung 40403, Taiwan; bao@mail.cmu.edu.tw; 10 Department of Surgery, Division of Urology, Chi-Mei Medical Center, Tainan 71004, Taiwan; s940854@gmail.com; 11 Department of Urology, Kaohsiung Municipal Ta-Tung Hospital, Kaohsiung 80145, Taiwan; 12 Taipei City Hospital Renai Branch, Taipei 106243, Taiwan; paichi.teng@gmail.com; 13 Institute of Cellular and System Medicine, National Health Research Institutes, Miaoli 350401, Taiwan; cpchuu@nhri.edu.tw; 14 Department Management Information Systems, National Pingtung University of Science and Technology, Pingtung 912301, Taiwan; dnchen@mail.npust.edu.tw; 15 Graduate Institute of Medicine, College of Medicine, Kaohsiung Medical University, Kaohsiung 80708, Taiwan; 16 Department of Medical Research, Kaohsiung Medical University Hospital, Kaohsiung 80756, Taiwan; 17 Ph.D. Program for Cancer Biology and Drug Discovery, China Medical University and Academia, Sinica 40403, Taiwan

**Keywords:** steroid hormone, prostate cancer, machine learning, prognostic signature

## Abstract

**Simple Summary:**

Prostate cancer (PC) is the second most common cancer worldwide and steroid hormone plays an important role in prostate carcinogenesis. Most patients with PC are initially sensitive to androgen deprivation therapy (ADT) but eventually become hormone refractory and reflect disease progression. The aim of the study was to investigate the genes which regulate the steroid hormone functional pathways and associate with the disease progression of PC. We identified a panel of eight-gene signatures that modulated steroid-hormone pathways and predicted the prognosis of PC using integrative bioinformatics analysis of multiple datasets validated from external cohorts. This panel could be used for predicting the prognosis of PC patients and might be associated with the drug response of hormonal therapies. Moreover, these genes in the signature could be potential targets to develop a novel treatment for castration-resistant PC therapy.

**Abstract:**

The importance of anti-androgen therapy for prostate cancer (PC) has been well recognized. However, the mechanisms underlying prostate cancer resistance to anti-androgens are not completely understood. Therefore, identifying pharmacological targets in driving the development of castration-resistant PC is necessary. In the present study, we sought to identify core genes in regulating steroid hormone pathways and associating them with the disease progression of PC. The selection of steroid hormone-associated genes was identified from functional databases, including gene ontology, KEGG, and Reactome. The gene expression profiles and relevant clinical information of patients with PC were obtained from TCGA and used to examine the genes associated with steroid hormone. The machine-learning algorithm was performed for key feature selection and signature construction. With the integrative bioinformatics analysis, an eight-gene signature, including *CA2*, *CYP2E1*, *HSD17B*, *SSTR3*, *SULT1E1*, *TUBB3*, *UCN*, and *UGT2B7* was established. Patients with higher expression of this gene signature had worse progression-free interval in both univariate and multivariate cox models adjusted for clinical variables. The expression of the gene signatures also showed the aggressiveness consistently in two external cohorts, PCS and PAM50. Our findings demonstrated a validated eight-gene signature could successfully predict PC prognosis and regulate the steroid hormone pathway.

## 1. Introduction

In 2020, prostate cancer (PC) was the second most frequent cancer and the fifth leading cause of cancer death among males worldwide, with an estimated 1.4 million newly diagnosed cases, causing approximately 374,000 deaths [1]. Steroid hormones, particularly androgen, play an important role in not only the development of benign prostatic hyperplasia, but the stimulation of prostate cancer growth as well [2]. The mutated oncogenes appear to play a role in the modulation of androgen response, although the exact genes have not been comprehensively defined [3].

The strategy of treating prostate cancer is based on the risk group. For lower-risk patients, localized therapy, including surgery or radiotherapy is recommended. For higher-risk or metastatic group, androgen deprivation therapy (ADT) plays a major key role in the treatment [4]. Many patients initially sensitive to ADT eventually become castration-resistant PC (CRPC), leading to disease progression and cancer death. It is believed that CRPC results from a failure of ADT to maintain durable suppression of androgen receptor (AR), which is a ligand-activated nuclear transcription factor belonging to the steroid hormone receptor family and the molecular target of ADT. In the recent decade, second-generation hormonal therapies such as abiraterone [5], apalutamide [6], enzalutamide [7], and darolutamide [8] have been developed to re-target the AR and have significantly improved the survival of PC patients. Randomized phase 3 clinical trials of abiraterone with steroid use have also demonstrated the improvement of treatment outcome in metastatic PC [9]. However, resistance to the second-generation AR antagonist has become challenging, and mechanisms underlying prostate cancer resistance to anti-androgens are not well understood.

Prostate cancer is often associated with somatic mutations that occur in the tumor, with an estimated 89% of metastatic CRPC tumors containing a potentially actionable mutation [10]. These mutations have the potential to be the target site for precision therapy. With the revolution of next-generation sequencing (NGS) technique, large sequencing datasets such as The Cancer Genome Atlas (TCGA) and the International Cancer Genome Consortium (ICGC) have been accomplished to provide human genomics data that has helped researchers discover cancer driver mutation genes and their regulating pathways [11,12]. For example, DNA damage response and repair (DDR) genes, including *BRCA1*, *BRCA2*, *CDK12*, *ATM*, *FANCD2*, and *RAD51C* were identified in 19% of tissue samples from 333 PC patients collected in a TCGA dataset [13]. Olaparib, a poly-(adenosine diphosphate-ribose) polymerase (PARP) inhibitor that mitigates tumor progression and improves survival, was approved as the first targeted therapy for PC patients with certain DDR gene mutations [14].

To identify the targeted genes related to cancer progression, Cox proportional hazard regression is traditionally well-accepted as the standard algorithm for survival analysis. However, in these high-throughput datasets, the number of variables is substantially higher than the number of samples. Compared to traditional regression statistics, machine learning (ML) algorithm has shown the ability to detect the key features predicting cancer prognosis from these complex datasets [15], and although ML has been applied to detect prognostic factors of PC in a few studies [16], none of them have focused on the genes in regulating the steroid hormone pathways, which are very important for PC morphology and progression. In the present study, we aimed to identify steroid hormone pathway-associated genes contributing to the progression and survival of PC by analyzing the data in several datasets using ML algorithm. In addition, the identified gene signature was further validated through external PC cohorts and analyzed using functional annotation to illustrate the regulatory pathways of these key steroid hormone-associated genes. These findings will help us more understand the underlying mechanism of CRPC and provide a potential target to develop specific therapies.

## 2. Materials and Methods

### 2.1. Data Collection and Preparation

The processed RNA sequencing (RNA-seq) profile and the corresponding clinical data of patients were curated from the DriverDB database [17,18,19]. Briefly, RNA-seq data of primary tumor (TP) and normal tissue (NT) in DriverDB were retrieved from the TCGA data portal in 1 January 2018 (https://portal.gdc.cancer.gov/) and genes were annotated with ENSEMBLE v91 [18]. A total of 20,495 genes with expression values Reads Per Kilobase per Million (RPKM) were curated. Patient’s survival data, including progression-free interval (PFI) and overall survival (OS), was curated from the TCGA Clinical Data Resource [12] (downloaded from: https://www.cell.com/cms/10.1016/j.cell.2018.02.052/attachment/bbf46a06-1fb0-417a-a259-fd47591180e4/mmc1.xlsx in 1 January 2018). The RNA-seq profiles, clinical data and survival data were matched according to the TCGA barcode. Only patients with prostate adenocarcinoma (PRAD) were retrieved for further analysis.

### 2.2. Steroid Hormone-Related Gene Selection

The keyword, “steroid hormone,” was used to match the pattern occurring in the pathway name or ontology term in 3 annotation databases: Gene Otology (GO) [20], Kyoto Encyclopedia of Genes and Genomes (KEGG) [21], and Reactome [22]. There were 19 steroid-hormone-relevant terms/pathways found, and the genes belonging to these terms/pathways were curated.

### 2.3. Differentially Expression Analysis

DEseq2 [23] was used to explore the gene that was differentially expressed between TP and NT in the PRAD subgroup. In the study, the significant differentially expressed (DE) gene was defined as the gene with an absolute log_2_ fold-change value greater than 1 and adjusted *p*-values, calculated by DEseq2, smaller than 0.05.

### 2.4. Survival Analysis

Both univariate and multivariate survival analyses were conducted under the R environment with Survival library package [24]. Two survival endpoints, PFI and OS, were analyzed. Gene expression values were normalized by the z-transformation method, mean value subtracting, and standard deviation scaling, with the normalized expression profiles being used in the following analysis. The relationship between survival probability and gene expression was illustrated by the Cox proportional hazards (CoxPH) model. Covariate variables, including (1) patient’s age at diagnosis (year, a continuous variable), (2) prostate-specific antigen (ng/mL, a continuous variable), (3) Gleason score divided by 2 groups (smaller than 7 vs. greater than 8, a categorical variable), and (4) TNM stage divided by 2 groups (T1/T2N0M0 vs. others, a categorical variable), were considered as confounding factors in the multivariate Cox proportional hazards model. The difference between groups in the probability of an event was tested by the log-rank test, and Kaplan–Meier survival curves were plotted.

### 2.5. Feature Selection and Signature Construction

Lasso (Least Absolute Shrinkage and Selection Operator), was a combination of ridge regression and was used to select gene feature [25,26]. A popular ML algorithm, the R-package “glmnet,” was used for feature (gene) selection and (gene) signature construction in this study. The linear combination of selected gene expression values weighted by the corresponding Lasso coefficients was used to construct the gene signature score:

$$\sum_{i=1}^{8}Lasso\_coefficient_{i}*Normalized\_Expression\_value_{i}$$

### 2.6. Independent Datasets Validation

The expression of the construct gene signature was examined with external cohorts. Two PC classifiers, Prostate Cancer Classification System (PCS) and PAM 50, were used from the public Prostate Cancer Transcriptome Atlas (PCTA, http://www.thepcta.org/; accessed on 1 December 2021) platform.

### 2.7. Functional Annotation

The functions of each gene in the gene signature were annotated through our in-house pipeline adopted in DriverDB database. In detail, function names in GO-BP (MSigDB-C5), pathway names in KEGG, and reaction names in REACTOME, which contained more than or equal to 3 of 8 candidate genes (Supplemental Table S1) were downloaded and searched. Then, the major regulatory pathways observed from GO-BP, KEGG, and REACTOME databases were identified and summarized in 2 scopes: steroid hormone-specific functions or all functions to explore potential interactions among candidate genes at the functional level.

## 3. Results

### 3.1. Identification of Steroid Hormone Genes Associated with Disease Progression in Prostate Cancer

**Figure 1:**
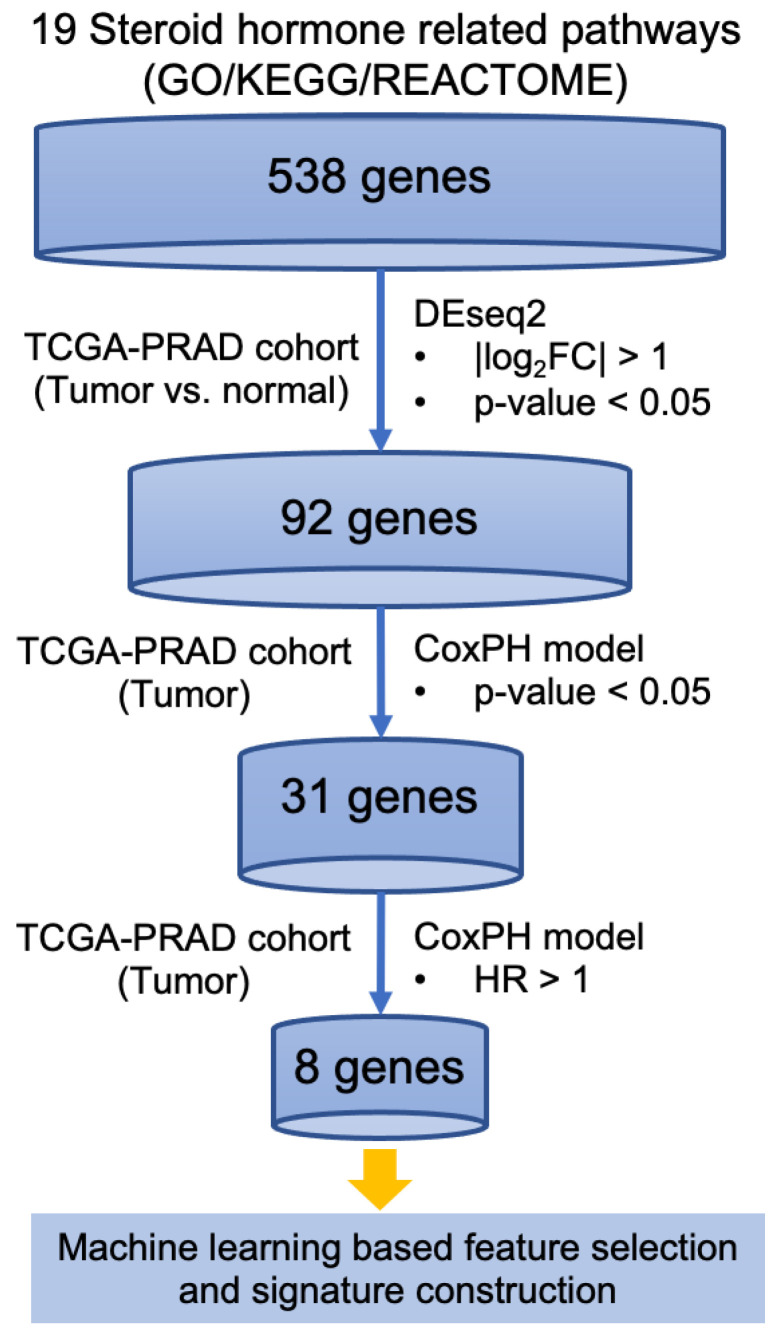
The signature exploration workflow. The workflow of generating the 8-gene signature associated with steroid hormone and prostate cancer progression.

In order to identify the steroid hormone genes associated with disease progression, we conducted the project with the assistance of PICOT tool (Table S2) and developed an integrated bioinformatics pipeline (Figure 1). Firstly, genes associated with the pathway matching the keyword “steroid hormone” in GO, KEGG, and Reactome databases were examined. A total of 538 genes associated with 19 steroid hormone-related pathways in these three databases were identified (Supplemental Table S3). Secondly, the transcriptomic profiling of these 538 genes was analyzed using RNA-seq data imported from DriverDBv3. Individual expressions of the steroid hormone-related 538 genes were examined. Finally, the expression levels were considered significantly different if the absolute value of log_2_ fold change was greater than 1 with the *p*-value less than 0.05, between TP and NT. After differential expression analysis, 92 significantly differentially expressed (DE) genes were identified (Supplemental Table S4).

To identify the genes associated with PC progression, the univariate CoxPH model was conducted to analyze each gene in the aspect of 5-year PFI among 497 patients from TCGA-PRAD cohort, respectively. PFI is an endpoint including cancer progression or cancer death. Two of 497 patients were excluded given incompatible gene expression levels, while 495 patients were stratified according to the median RNA expression levels. Genes with a *p*-value smaller than 0.05 were considered to be significantly related with the patient’s 5-year PFI. Among 92 DE genes, a total of 31 genes were found to be related to clinical prognosis and 8 genes were identified as the genes associated with poor clinical outcome (hazard ratio > 1), with these oncogenes showing positive association with the disease progression. As a consequence, these genes may serve as targets to improve outcome of PC [27]. The corresponding data of the eight genes, including *p*-value and hazard ratio (HR) of PFI, are shown in Table 1.

### 3.2. Identification of an Eight-Gene Signature Predicting PC Survival

**Figure 2:**
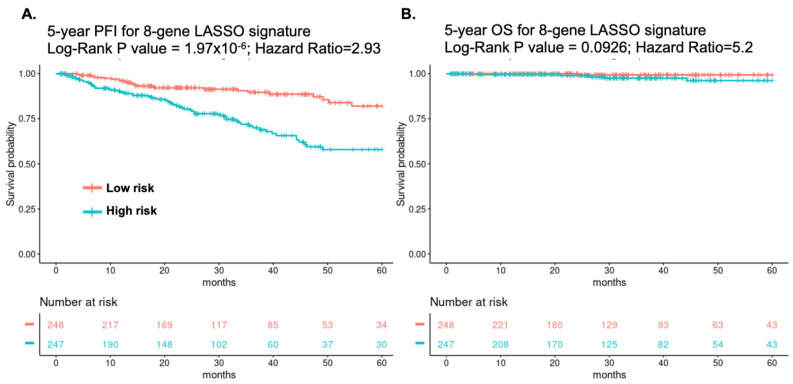
Progression-free interval (PFI) and overall survival (OS). Kaplan–Meier curves for (**A**) 5-year progression-free interval and (**B**) 5-year overall survival of the 8-gene signature. Patients were dichotomized into the “Low risk” group and the “High risk” group according to the 8-gene signature scores. The number of patients of the two risk groups in different following time in month were shown in the bottom tables of KM plots, respectively.

To clarify the most key genes associated with the clinical prognosis of PC, Lasso regression analysis, which is one of the popular ML algorithms, was performed for the feature selection and coefficient training. Consequently, a panel of eight steroid hormone-related genes, including *CA2*, *CYP2E1*, *HSD17B*, *SSTR3*, *SULT1E1*, *TUBB3*, *UCN*, and *UGT2B7* was selected, and the corresponding Lasso coefficients were estimated. The linear combination of eight gene expression values weighted by the corresponding Lasso coefficients was used to construct the eight-gene signature score. Patients were stratified into two groups, a high-risk group (*n* = 248) and a low-risk group (*n* = 247), according to the median gene-signature score. The eight-gene signature was evaluated by the univariate CoxPH model, and the prognostic ability of the 8-gene signature was evaluated by the log-rank test. The correspondent Kaplan–Meier plot of the patients from TCGA-PRAD database demonstrated that the patients with higher expression of eight-gene signature had significantly worse 5-year PFI compared to those with lower expression (HR = 2.93, 95% CI = 1.84–4.67, *p* < 0.001, Figure 2A), while the patients with lower expression of the eight-gene signature also had marginal significantly better 5-year overall survival (OS) compared to those with higher expression (HR = 5.2, 95% CI = 0.61–44.54, *p* = 0.093 Figure 2B).

### 3.3. Multivariate Cox Regression Analysis with Clinical Variables

**Figure 3:**
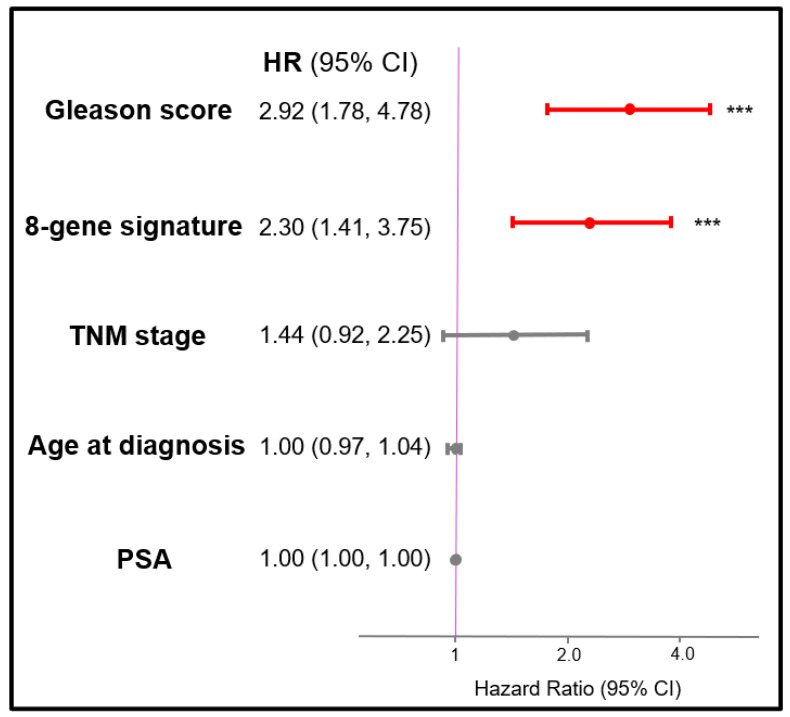
Multivariate analysis for progression-free interval. Multivariate Cox regression of the 8-gene signature with clinical variables. Significance levels are annotated. Clinical factors such as gleason score, psa level, tumor TNM stage, and age at diagnosis were considered as confounding variables in the analysis. Both hazard ratios and 95% confidence intervals were shown in the forest plot and factors reached a significant level were plotted in red. *** *p*-value < 0.001.

A multivariate CoxPH model was conducted to adjust possible clinical confounding variables, including age, baseline prostate-specific antigen (PSA) level, Gleason score, and TNM staging. As shown in Figure 3, the patients with higher expression of eight-gene signature remained independently associated with poor PFI (HR = 2.30, 95% CI = 1.41–3.75, *p* < 0.001). The higher Gleason scores (>7) also had significantly worse PFI compared to lower Gleason score (HR = 2.92, 95% CI = 1.78–4.78, *p* < 0.001). Other parameters, i.e., age, PSA level, and TNM staging, did not reach statistical significance in this model.

### 3.4. Expression of the Eight-Gene Panel Based on External PC Cohort’s Validation

**Figure 4:**
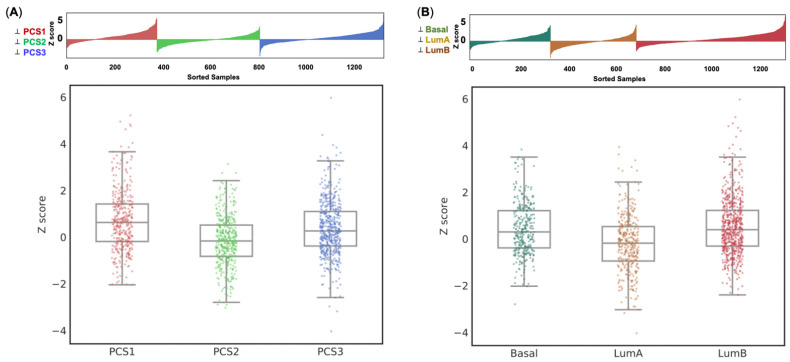
External validation. The expression of the 8-gene signature based on (**A**) PCS subtypes and (**B**) PAM50. The distributions of z-score transformed expression values in each group are shown in lollipop plot (**top**) and box plot (**bottom**). Higher expression of 8-gene signature in both aggressive subtypes (PCS1 and LumB) of two independent cohorts (PCS and PAM50) demonstrated the consistent results in external validation.

In this study, we examined the expression of identified eight-gene panel based on these two well-known PC classifiers, PCS and PAM50, using data from an independent cohort [28]. Prostate Cancer Classification System (PCS) is a validated classification system that classifies PC into three distinct subtypes (PCS1, PCS2, and PCS3) based on a 37-gene panel [28], whereas PAM 50 is another 50-gene panel used to categorize PC into basal, luminal A and luminal B subtypes [29]. In short, PCS1 tumors are the most aggressive type in PCS, while Luminal B is the most aggressive type in PAM50. The analysis of gene expression based on PAM50 and PCS subtypes was conducted using the public Prostate Cancer Transcriptome Atlas (PCTA) platform. The Z scores of our eight-gene panel were significantly associated with PCS (one-way ANOVA test F value = 15.547, *p* < 0.001; Ranksums test fold change = 0.385, *p* < 0.001, Figure 4A) and PAM50 (one-way ANOVA test F value = 15.176, *p* < 0.001; Rank-sums test Fold change = 0.22, *p* = 0.023, Figure 4B) subtypes. In terms of the PAM50 system, luminal B had the highest Z scores. In the PCS system, PCS1 had the highest Z scores. To sum up, the expression of our eight-gene signature was consistent with the aggressiveness regarding either PAM50 or PCS system.

### 3.5. Functional Annotation of the Steroid Hormone Genes Associated with Prognosis

**Figure 5:**
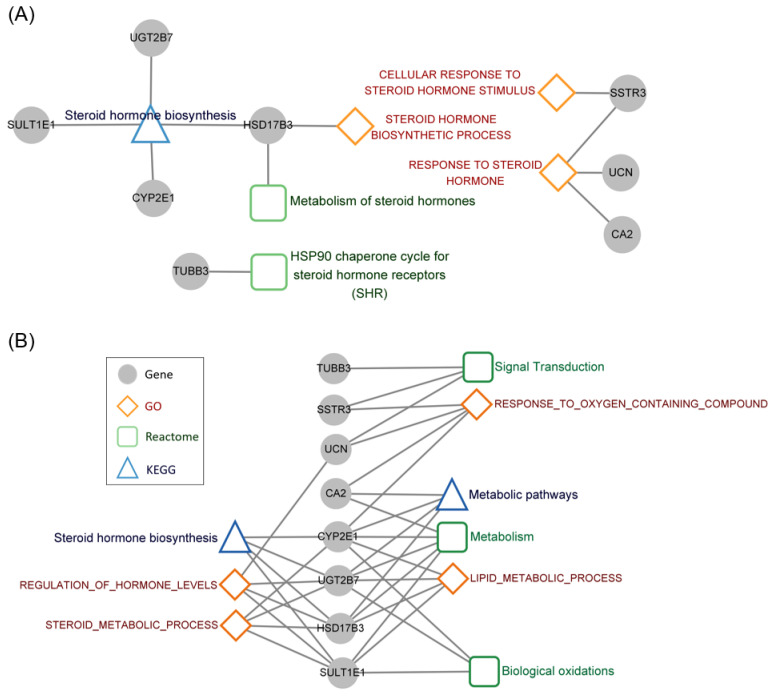
The regulatory pathways. Functional annotation of 8 genes based on three databases in aspects of (**A**) steroid hormone-specific and (**B**) all functions containing more than 3 of 8 signature genes. The gene is illustrated as a filled grey circle. Databases are drawn as an empty triangle, rectangle, and diamond. The grey edge represents linkage between annotated gene and the corresponding function.

As shown in Figure 5A, the identified 8 genes, *CA2*, *CYP2E1*, *HSD17B*, *SSTR3*, *SULT1E1*, *TUBB3*, *UCN*, and *UGT2B7* were involved in regulating “steroid hormone biosynthesis” in the KEGG database; “metabolism of steroid hormones” and “HSP 90 chaperone cycle for steroid hormone receptors” in Reactome; and “cellular response to steroid hormone stimulus”, “steroid hormone biosynthetic process” and response to steroid hormone in GO. In order to elucidate the crucial functions regulated by the identified eight genes, functional annotation was performed using GO, KEGG, and Reactome pathway databases. Only the pathways regulated simultaneously by at least 3 genes among the 8-gene signature were regarded as key functions. The cross interaction of these 8 genes among KEGG, Reactome, and GO databases is shown in Figure 5B. Besides “steroid hormone” related pathways, the identified 8 genes also cross-linked with “metabolic pathways”, “signal transduction,” “metabolism,” “biological oxidations,” “response to oxygen containing compound” and “lipid metabolic process.”

## 4. Discussion

Steroid hormone receptors, especially AR, provide a critical pathway for PC progression, and ADT remains the backbone for PC treatment. However, it has been revealed that genetic aberrations result in the resistance of ADT, through either androgen-dependent or androgen-independent mechanisms [30]. The first gene pathway-based therapy for PC, i.e., olaparib, was just approved in May 2020; consequently, there is a clinically unmet need for genomic-based biomarker and pharmacological targeting for PC. Krebs et al. discovered the downregulation of miRNAs (i.e., miR-221-3p) expression regulating VEGFR2 expression, predicting the prognosis of high-risk PC and the response to tyrosine kinase inhibitors [31]. In our study, we identified an eight-gene signature, which is related to the steroid hormone pathways and predicts the clinical prognosis of PC. The eight genes in this signature were *CA2*, *CYP2E1*, *HSD17B*, *SSTR3*, *SULT1E1*, *TUBB3*, *UCN,* and *UGT2B7,* respectively. Among these eight genes, five genes, including *HSD17B*, *SSTR3*, *SULT1E1*, *TUBB3,* and *UGT2B7,* were identified to be associated with both PC prognosis and hormone refractoriness previously. HSD17B3, which is the key enzyme for the metabolism of progestins to adrenal androgens and subsequent conversion to testosterone, is significantly upregulated in castration-resistant metastases compared to untreated PC [32,33]. Among the analogs of peptide hormones, somatostatin (SST) analogs were found to decrease tumor cell growth and angiogenesis and increase cancer cell apoptosis. SSTR3, which is one of the somatostatin receptor (SSTR) families, was identified as changing the expression in the membrane components in hormone-refractory PC compared with hormone-sensitive PC [34]. The overexpression of SSTR3 in CRPC patients detected by PET/CT was also reported [35]. Estrogen sulfotransferase (SULT1E1) belongs to the cytosolic sulfotransferase superfamily, which are Phase II drug-metabolizing enzymes. SULT1E1 catalyzes the sulfation of estrogens, which play a vital regulatory role in the development and propagation of reproductive malignancies such as breast and prostate cancer [36]. Six single nucleotide polymorphisms (SNPs) in *SULT1E1* were identified to be associated with time to treatment failure (TTF) in 68 patients with CRPC under the treatment of abiraterone [37]. Tubulin-β3 encoded by the *Tubulin-β3* (*TUBB3*) gene is one of the seven β-microtubule proteins normally expressed in neuronal cells and testis. TUBB3 has been reported to be associated with phosphatase and tensin homolog (PTEN) and neuroendocrine differentiation, which might induce an aggressive type of PC [38]. The expression of TUBB3 is reported to be not only associated with the progression of CRPC in a study enrolling 138 human prostate tumor specimens [39] but is also able to predict the treatment response to taxene-based chemotherapy for CRPC [40]. The *UGT2B7* gene, belonging to UDP-glucuronosyltransferase (UGT) enzymes, is known particularly for its wide spectrum of specificity for all classes of steroids, such as conjugating (1) 5a-reduced metabolites of mineralocorticoids, glucocorticoids, progestins and androgens, and (2) 5b-reduced C21 and C19 steroids [41]. The role of UGT2B7 to expedite the progression of CRPC has been noticed by the mechanism of promoting ligand-independent AR signaling [42]. In the remaining three genes, two of them, *CYP2E1* and *UCN*, have shown the association with prostate carcinogenesis only but revealed no clear role of the prognosis of PC receiving ADT. The differential expression of urocortin (UCN) between prostate malignancy and normal tissue has been indicated [43,44]. The polymorphisms of the *CYP2E1* gene might be associated with a two-fold increased risk for the development of PC [45]. Lastly, carbonic anhydrase II (CA2) is one of 16 forms of human α carbonic anhydrase and has been shown to be upregulated or associated with androgen receptors in various cancer types such as meningioma [46] and breast cancer, but not in PC [47]. To sum up, this previous literature confirms the feasibility of our gene candidates and the potential for further research.

Although the univariate cox analyses revealed statistical significance of our eight-gene signature in terms of PFI, there may be clinical confounding variables that led to bias in the analyses. To overcome this issue, a multivariate Cox regression model adjusting for commonly seen clinical variables in PC was conducted. These clinical variables, including age, TNM staging, PSA at diagnosis, and Gleason score, were selected given clinical practice guidelines using these four factors together to stratify the subgroup of PC and further different treatments [4]. The Gleason score and TNM staging showed significant relationship to PFI, whereas age and PSA did not in the univariate cox PH model in our study (Supplemental Table S5). Old age at prostate cancer diagnosis has been reported to be associated with poor prognosis in several observational studies, but some argue the association might be explained by less treatment with curative intent rather than disease aggressiveness [48]. Baseline PSA has been introduced to assist PC diagnosis and management for decades, but considered to be inappropriate as a predictive biomarker alone in recent research [49]; accordingly, our eight-gene signature, possessing statistical significance in multivariate analysis, could be considered as improving clinical diagnosis and management of this disease with these four traditional clinical factors.

External cohort validation is needed for bioinformatics analysis. Two well-validated classifiers (PCS and PAM50) were used in our study. PCS is a classification system derived from a large cohort (*n* = 1321) of human PC transcriptome profiles from 38 distinct cohorts [28]. Analysis of subtype-specific gene expression patterns showed that PCS1 and PCS2 tumors reflect luminal subtypes, while PCS1 tumors progress more rapidly in comparison with PCS2 or PCS3. PAM50 is a 50-gene panel that classifies breast cancer into five intrinsic molecular subtypes and has become the basis for commercial testing of breast cancer [50]. Furthermore, the subtypes of PAM 50 also displayed significant differences in prognosis and response to other cancers, including PC, which is also a sex hormone-derived cancer [29,51]. Zhao et al. successfully applied PAM 50 into a PC cohort including 3782 samples and identified luminal B tumors exhibiting the poorest clinical outcomes on both univariate and multivariate analyses [30]. The expressions of our eight-gene panel highly correlated with aggressiveness in both PCS and PAM50 classifiers. This result enables the significance of our gene signature to be more convincing.

Functional annotation has been widely applied on analyzing the biological processes of collecting genes based on molecular function, biological role, subcellular location, and the regulatory pathways [52,53]. The functional annotation results of the eight-gene signature pointed out that the cross-reaction of identified eight genes were involved in regulating steroid hormone biosynthesis and process and modulating the cellular response of steroid hormone as well as affecting the signal transduction, biological oxidation, and metabolic pathways. PC resistance to androgen deprivation therapies ensues when tumors engage metabolic processes that produce sustained androgen levels in the tissue. Ablation of UGT enzymes (UGT2B15 and UGT2B17) has been demonstrated to increase free dihydrotestosterone restoration, sustain androgen signaling, and develop castration resistance [54]. Although the role of UGT2B7 has not been extensively investigated, UGT2B7 has been reported to exhibit high activity of steroid glucuronidation and is considered as a major enzyme responsible for the conjugation of androgens in humans [55]. Our results showed that a decrease of *UGT2B7* expression is associated with the poor prognosis of PC, suggesting that *UGT2B7* might play a critical role in driving castration resistance.

## 5. Conclusions

In conclusion, the mechanisms regarding PC resistance to anti-androgens are incompletely understood, thus the novel and precisely targeted therapy for CRPC is still in demand. Using the ML-based bioinformatics analysis, we successfully identified an eight-gene signature consisting of *CA2*, *CYP2E1*, *HSD17B*, *SSTR3*, *SULT1E1*, *TUBB3*, *UCN,* and *UGT2B7*, predicting the prognosis of PC. These signature clinical and biological associations are consistent among multiple datasets, including TCGA, DriverDBv3, PCS, PAM50 GO, KEGG, and Reactome. These genes not only regulate the pathways related to steroid hormone but modulate the function of metabolism and signal transduction as well. This eight-gene signature could become a promising panel of biomarkers to screen the prognosis of PC patients and might be pharmacological targets to develop therapies for CRPC. Further large prospective cohort studies are required for further validation, and additional experimental studies in vivo are also needed to provide robust evidence of the role of anti-cancer therapy.

## Figures and Tables

**Table 1:** The differential expression and hazard ratio (HR) of progression-free interval (PFI). The log_2_ fold change and corresponding HR of PFI of each of the 8 genes.

	Differential Expression Analysis		Survival Analysis (PFI)		
Gene	Log2 Fold Change	Adjusted *p*-Value	HR	CI95	*p*-Value
*CA2*	−4.48699	2.20 × 10^−78^	2.14	1.36–3.37	0.001038
*CYP2E1*	−1.88521	1.31 × 10^−25^	1.55	1.01–2.38	0.043481
*HSD17B3*	1.32350	4.89 × 10^−11^	2.19	1.40–3.40	0.000527
*SSTR3*	−1.21147	2.44 × 10^−5^	1.83	1.18–2.83	0.006554
*SULT1E1*	−1.23635	9.17 × 10^−6^	1.94	1.24–3.01	0.003371
*TUBB3*	1.32113	2.14 × 10^−10^	2.27	1.45–3.54	0.000319
*UCN*	2.16915	4.31 × 10^−41^	1.94	1.24–3.01	0.003137
*UGT2B7*	−5.67669	1.25 × 10^−54^	1.64	1.07–2.52	0.023815

## Data Availability

Raw data could be obtained from TCGA portal and results of analysis could be found in the Supplemental data of this paper.

## Supplementary Materials

The following supporting information can be downloaded at: https://www.mdpi.com/article/10.3390/cancers14061565/s1, Table S1: Functional annotations of genes in MsigDB-C5, KEGG, and REACTOME. Table S2: The PICOT analysis of the study. Table S3: Steroid hormone relevant pathways in 3 databases, Table S4: The log_2_ fold change and corresponding HR of PFI for 92 significantly differentially expressed (SDE) genes, Table S5: Univariate analysis results of 8-gene signature and clinical variables.
