# Supplementary material for: Identification of a Steroid Hormone-Associated Gene Signature Predicting the Prognosis of Prostate Cancer through an Integrative Bioinformatics Analysis
Source: Cancers (Basel). 2022 Mar 19;14(6):1565. doi: 10.3390/cancers14061565 (PMC8946240; doi:10.3390/cancers14061565)
Supplement: Supplementary file 1 [file cancers-14-01565-s001.zip › cancers-1594319-supplementary.pdf]

Supplementary Materials:

Identification of a Steroid Hormone-Associated Gene Signature Predicting the Prognosis of Prostate Cancer Through an Integrative Bioinformatics Analysis

Yo-Liang Lai [1,2], Chia-Hsin Liu [3], Shu-Chi Wang [4], Shu-Pin Huang [5,6,7,8], Yi-Chun Cho [3], Bo-Ying Bao [9], Chia-Cheng Su [10], Hsin-Chih Yeh [5,11], Cheng-Hsueh Lee [6], Pai-Chi Teng [12], Chih-Pin Chuu [13], Deng-Neng Chen [14], Chia-Yang Li [15,16,*] and Wei-Chung Cheng [1,2,17,*]

Table S1. Functional annotations of genes in MsigDB-C5, KEGG, and REACTOME.

Function name	Gene_num.	DB
Lipid metabolic process	4	GO-BP
Regulation of hormone levels	4	GO-BP
Response to oxygen-containing compound	4	GO-BP
Steroid metabolic process	4	GO-BP
Cell-cell signaling	3	GO-BP
Cellular hormone metabolic process	3	GO-BP
G protein-coupled receptor signaling pathway	3	GO-BP
Hormone metabolic process	3	GO-BP
Organic acid metabolic process	3	GO-BP
Organic hydroxy compound metabolic process	3	GO-BP
Oxidation regulation process	3	GO-BP
Reproduction	3	GO-BP
Response to endogenous stimulus	3	GO-BP
Response to hormone	3	GO-BP
Response to lipid	3	GO-BP
Response to organic cyclic compound	3	GO-BP
Response to steroid hormone	3	GO-BP
Androgen metabolic process	2	GO-BP
Apoptotic process	2	GO-BP
Cell projection organization	2	GO-BP
Cellular response to endogenous stimulus	2	GO-BP
Cellular response to hormone stimulus	2	GO-BP
Cellular response to oxygen-containing compound	2	GO-BP
Developmental process involved in reproduction	2	GO-BP
Ion transport	2	GO-BP
Lipid biosynthetic process	2	GO-BP
Monocarboxylic acid metabolic process	2	GO-BP
Multi-organism process	2	GO-BP
Neurogenesis	2	GO-BP

Neuron development	2	GO-BP
Neuron differentiation	2	GO-BP
Neuropeptide signaling pathway	2	GO-BP
Positive regulation of cell differentiation	2	GO-BP
Positive regulation of developmental process	2	GO-BP
Positive regulation of multicellular organismal process	2	GO-BP
Positive regulation of signaling	2	GO-BP
Positive regulation of transport	2	GO-BP
Regulation of cell differentiation	2	GO-BP
Regulation of ion transport	2	GO-BP
Regulation of peptide transport	2	GO-BP
Regulation of transport	2	GO-BP
Regulation of trans synaptic signaling	2	GO-BP
Response to abiotic stimulus	2	GO-BP
Response to corticosteroid	2	GO-BP
Response to estradiol	2	GO-BP
Response to extracellular stimulus	2	GO-BP
Response to inorganic substance	2	GO-BP
Response to nitrogen compound	2	GO-BP
Response to oxidative stress	2	GO-BP
Secretion	2	GO-BP
Small molecule biosynthetic process	2	GO-BP
Synaptic signaling	2	GO-BP
Metabolic pathways	4	KEGG
Steroid hormone biosynthesis	4	KEGG
Bile secretion	2	KEGG
Chemical carcinogenesis	2	KEGG
Drug metabolism-cytochrome P450	2	KEGG
Drug metabolism-other enzymes	2	KEGG
Metabolism of xenobiotics by cytochrome P450	2	KEGG
Neuroactive ligand-receptor interaction	2	KEGG
Metabolism	5	REACTOME
Biological oxidations	3	REACTOME
Signal transduction	3	REACTOME
Cilium assembly	2	REACTOME
GPCR ligand binding	2	REACTOME
Metabolism of lipids	2	REACTOME
Metabolism of proteins	2	REACTOME
Organelle biogenesis and maintenance	2	REACTOME
Phase II-conjugation of compounds	2	REACTOME
Signaling by GPCR	2	REACTOME

Table S2. The PICOT analysis of the study.

P (Patient/Population)	I (Issue/Intervention)	C (Comparison)	O (Outcome)	T (Time Frame)
Patients with prostate cancer	Steroid hormone - associated gene signature	Higher expression versus lower expression of the signature	Disease progression and patient survival	5 years

Table S3. Steroid hormone relevant pathways in 3 databases

Database	Pathway name
GO	Response to steroid hormone
GO	Cellular response to steroid hormone stimulus
GO	Steroid hormone mediated signaling pathway
GO	Steroid hormone receptor activity
GO	C21 steroid hormone biosynthetic process
GO	C21 steroid hormone metabolic process
GO	Steroid hormone biosynthetic process
GO	Steroid hormone secretion
GO	Intracellular steroid hormone receptor signaling pathway
GO	Positive regulation of intracellular steroid hormone receptor signaling pathway
GO	Regulation of intracellular steroid hormone receptor signaling pathway
GO	Steroid hormone receptor binding
GO	Negative regulation of intracellular steroid hormone receptor signaling pathway
GO	Regulation of steroid hormone biosynthetic process
GO	Steroid hormone binding
GO	Positive regulation of steroid hormone secretion
KEGG	Steroid hormone biosynthesis
REACTOME	HSP90 chaperone cycle for steroid hormone receptors (SHR)
REACTOME	Metabolism of steroid hormones

Table S4. The $\log_2$ fold change and corresponding HR of PFI for 92 significantly differentially expressed (SDE) genes.

gene_symbol	id	log2 fold change	pvalue	padj	mean_rpkm
CA2	ENSG00000104267	-4.49	2.20E-78	2.78E-75	75354.21
CYP2E1	ENSG00000130649	-1.89	1.31E-25	6.61E-24	7413.98
HSD17B3	ENSG00000130948	1.32	4.89E-11	3.69E-10	7951.94
SSTR3	ENSG00000278195	-1.21	2.44E-05	8.00E-05	596.64
SULT1E1	ENSG00000109193	-1.24	9.17E-06	3.22E-05	2365.44
TUBB3	ENSG00000258947	1.32	2.14E-10	1.48E-09	5017.87
UCN	ENSG00000163794	2.17	4.31E-41	9.53E-39	54606.50
UGT2B7	ENSG00000171234	-5.68	1.25E-54	5.80E-52	6311.85
CD38	ENSG00000004468	-1.52	1.53E-08	8.15E-08	222769.80
CALCR	ENSG00000004948	-1.08	1.77E-07	8.08E-07	379.93
ATP1A2	ENSG00000018625	-1.67	1.89E-15	2.50E-14	141877.27
SOX30	ENSG00000039600	-1.04	1.06E-09	6.64E-09	2853.43
LMO3	ENSG00000048540	-2.22	4.27E-35	5.79E-33	22299.63
DKK3	ENSG00000050165	-1.09	4.87E-14	5.35E-13	393629.44
TP63	ENSG00000073282	-1.85	1.11E-15	1.51E-14	98403.31
PTGS2	ENSG00000073756	-3.06	5.26E-39	9.96E-37	307510.42
PGR	ENSG00000082175	-1.55	1.92E-21	5.65E-20	16511.80
GSTP1	ENSG00000084207	-2.10	1.82E-36	2.86E-34	1516068.57
AKR1B1	ENSG00000085662	-3.92	2.68E-128	1.90E-124	338075.19
HSD17B2	ENSG00000086696	-1.14	4.20E-06	1.56E-05	2152.50
BMP7	ENSG00000101144	-1.01	3.07E-07	1.36E-06	65907.03
CAV1	ENSG00000105974	-1.76	8.90E-35	1.16E-32	464839.66
CYP3A5	ENSG00000106258	-2.05	2.29E-18	4.42E-17	33260.10
ALDH3A1	ENSG00000108602	-1.58	1.98E-14	2.28E-13	9043.21
AREG	ENSG00000109321	-1.66	3.61E-11	2.78E-10	47619.09
PPARGC1A	ENSG00000109819	-1.80	4.26E-24	1.78E-22	12164.83
ADTRP	ENSG00000111863	-3.20	4.00E-41	8.92E-39	19548.57
BMP5	ENSG00000112175	-2.24	8.87E-08	4.26E-07	39038.18
BCHE	ENSG00000114200	-1.84	1.06E-17	1.89E-16	39588.59
SDC1	ENSG00000115884	-1.02	1.62E-11	1.30E-10	799109.96
PADI2	ENSG00000117115	-1.04	1.64E-08	8.72E-08	33565.92
HSD11B1	ENSG00000117594	-1.41	1.65E-16	2.51E-15	67987.98
NR4A3	ENSG00000119508	-1.28	7.34E-07	3.07E-06	86517.65
TGFB3	ENSG00000119699	-1.21	3.40E-12	2.98E-11	303770.31
SCGB2A1	ENSG00000124939	-1.47	3.37E-13	3.35E-12	104030.49
PTGER2	ENSG00000125384	-1.04	4.36E-12	3.78E-11	67182.83
TUBA4A	ENSG00000127824	-1.04	3.80E-15	4.85E-14	200297.01
FOXP2	ENSG00000128573	-1.32	8.91E-17	1.40E-15	6401.57
CDO1	ENSG00000129596	-2.31	4.21E-21	1.18E-19	169506.05

ASS1	ENSG00000130707	-1.25	6.43E-17	1.03E-15	677936.22
FSHB	ENSG00000131808	1.21	0.000757922	0.001913579	453.49
DNAJB1	ENSG00000132002	-1.21	2.42E-20	6.10E-19	1224334.74
SERPINF1	ENSG00000132386	-1.03	6.63E-13	6.35E-12	735848.39
RERG	ENSG00000134533	-1.22	1.98E-25	9.70E-24	73669.95
ANXA1	ENSG00000135046	-1.05	4.24E-15	5.36E-14	733037.50
HTR1B	ENSG00000135312	-1.49	7.38E-10	4.73E-09	1559.16
IL1RN	ENSG00000136689	-1.48	1.09E-15	1.49E-14	22917.52
TUBB2A	ENSG00000137267	1.33	1.05E-18	2.12E-17	537419.28
TLR2	ENSG00000137462	-1.48	8.88E-26	4.60E-24	34667.45
MGARP	ENSG00000137463	-1.45	6.63E-24	2.69E-22	12761.91
METTL21C	ENSG00000139780	-1.54	0.003199539	0.007147305	4911.22
CYP11A1	ENSG00000140459	-1.72	3.87E-16	5.61E-15	5630.53
CYP1A2	ENSG00000140505	-2.08	1.89E-09	1.15E-08	677.95
NTRK3	ENSG00000140538	-1.16	7.38E-11	5.44E-10	4409.41
TGFB1I1	ENSG00000140682	-1.32	2.13E-18	4.12E-17	223371.80
RXRG	ENSG00000143171	-1.55	1.20E-12	1.11E-11	4560.43
AGTR1	ENSG00000144891	1.37	8.46E-08	4.07E-07	151191.85
STAR	ENSG00000147465	-1.02	2.00E-09	1.21E-08	1205.72
TACC1	ENSG00000147526	-1.06	6.94E-22	2.16E-20	259240.11
SCGB1A1	ENSG00000149021	-2.51	1.61E-13	1.66E-12	621470.70
AKR1C2	ENSG00000151632	-1.22	9.21E-10	5.82E-09	7737.45
PPARGC1B	ENSG00000155846	-1.26	4.13E-24	1.73E-22	11586.99
FBXO32	ENSG00000156804	-1.02	5.67E-25	2.63E-23	364922.30
DYNC1I1	ENSG00000158560	-1.25	6.28E-19	1.31E-17	33041.51
S100B	ENSG00000160307	-1.36	7.97E-11	5.85E-10	45842.68
CYP3A7	ENSG00000160870	-1.55	2.49E-16	3.71E-15	3338.68
CLDN1	ENSG00000163347	-1.06	7.34E-09	4.11E-08	254543.03
STARD4	ENSG00000164211	-1.14	1.32E-16	2.04E-15	61627.09
PRKCB	ENSG00000166501	-1.83	1.68E-25	8.33E-24	29926.74
GALR1	ENSG00000166573	-1.72	1.79E-12	1.62E-11	1756.95
UGT1A6	ENSG00000167165	-1.64	3.08E-09	1.81E-08	1280.68
OR51E2	ENSG00000167332	2.37	1.95E-23	7.45E-22	4734909.12
DDIT4	ENSG00000168209	-1.23	9.70E-19	1.97E-17	738027.89
FAM107A	ENSG00000168309	-1.63	7.62E-25	3.45E-23	212074.50
PAQR8	ENSG00000170915	-2.62	1.72E-76	1.80E-73	60586.61
BCL2	ENSG00000171791	-1.09	5.70E-16	8.04E-15	82910.20
C1QTNF1	ENSG00000173918	-1.67	1.54E-28	1.13E-26	179875.02
NPAS4	ENSG00000174576	-1.22	6.92E-08	3.37E-07	2961.82
FOSL1	ENSG00000175592	-1.65	2.03E-09	1.23E-08	63062.37
CAPZA3	ENSG00000177938	-3.28	8.72E-12	7.26E-11	387.69
SSTR2	ENSG00000180616	-1.28	5.57E-17	9.00E-16	6496.59
PAQR7	ENSG00000182749	-1.50	1.23E-43	3.17E-41	113748.65

PAPPA	ENSG00000182752	-1.03	3.19E-11	2.48E-10	4524.84
FOXL2	ENSG00000183770	2.34	8.37E-18	1.51E-16	16957.40
SLIT3	ENSG00000184347	-1.31	6.36E-17	1.02E-15	37665.25
KANK2	ENSG00000197256	-1.28	3.55E-28	2.53E-26	432224.65
TUBA3C	ENSG00000198033	-1.80	6.04E-08	2.97E-07	544.62
AKR1C4	ENSG00000198610	2.63	1.26E-05	4.32E-05	367.67
HSPA1A	ENSG00000204389	-1.06	1.57E-09	9.61E-09	1800080.53
AKR1B15	ENSG00000227471	-1.70	1.52E-05	5.13E-05	1166.38
NEFL	ENSG00000277586	-1.52	1.09E-05	3.78E-05	27563.60
SRD5A2	ENSG00000277893	-1.70	8.78E-18	1.58E-16	94036.35

Table S5. Univariate analysis results of 8-gene signature and clinical variables.

5-year PFI uni-variate CoxPH model	HR	P	L95	U95	C_index
age_at_diagnosis	1.02	0.13	0.99	1.06	0.55
tnm	1.86	0.00	1.21	2.86	0.58
gleason_2group	3.84	0.00	2.44	6.05	0.66
psa	1.00	0.35	1.00	1.00	0.33
8-gene signature	2.93	0.00	1.84	4.67	0.62
